# Cerebral relapse of metastatic gastrointestinal stromal tumor during treatment with imatinib mesylate: Case report

**DOI:** 10.1186/1471-2407-4-74

**Published:** 2004-10-09

**Authors:** Brett Hughes, Desmond Yip, David Goldstein, Paul Waring, Victoria Beshay, Guan Chong

**Affiliations:** 1 Medical Oncology Unit, The Canberra Hospital, Yamba Drive, Garran, ACT, Australia; 2 Institute of Oncology, Prince of Wales Hospital, Randwick, NSW, Australia; 3 Department of Pathology, Peter MacCallum Cancer Centre, Melbourne, VIC Australia; 4 Department of Surgery, The Canberra Hospital, Garran, ACT, Australia

## Abstract

**Background:**

The management of unresectable or metastatic gastrointestinal stromal tumors (GISTs) has previously been difficult as they are resistant to conventional chemotherapy and radiation. The development of imatinib mesylate has made a major impact on the management of advanced GISTs. It is apparent that there are sanctuary sites such as the central nervous system where imatinib does not achieve adequate concentrations. We describe the case of a man with metastatic GIST who experienced multiple cerebral relapses of disease while systemic disease progression appeared to be controlled by imatinib.

**Case presentation:**

A 47-year-old man presented in July 1999 with a jejunal GIST with multiple hepatic metastases. The jejunal primary was resected and after unsuccessful cytoreductive chemotherapy, the liver metastases were also resected in December 1999. The patient subsequently relapsed in August 2001 with symptomatic hepatic, subcutaneous gluteal, left choroidal and right ocular metastases all confirmed on CT and PET scanning. Biopsy confirmed recurrent GIST. MRI and lumbar puncture excluded central nervous system involvement. The patient was commenced on imatinib 400 mg bd in September 2001 through a clinical trial.

The symptoms improved with objective PET and CT scan response until December 2002 when the patient developed a right-sided foot drop. MRI scan showed a left parasagittal tumor which was resected and confirmed histologically to be metastatic GIST. Imatinib was ceased pre-operatively due to the trial protocol but recommenced in February 2003 on a compassionate use program. The left parasagittal metastasis recurred and required subsequent re-excision in September 2003 and January 2004. Control of the systemic GIST was temporarily lost on reduction of the dose of imatinib (due to limited drug supply) but on increasing the dose back to 800 mg per day, systemic disease was stabilized for a period of time before generalised progression occurred.

**Conclusion:**

This case illustrates that the brain can be a sanctuary site to treatment of GISTs with imatinib. Maintaining dosing of imatinib in the face of isolated sites of disease progression is also important, as other metastatic sites may still be sensitive.

## Background

Gastrointestinal stromal tumors (GISTs) are rare mesenchymal gastrointestinal tumors which can have an aggressive course. Management of these tumors apart from surgical resection has been difficult in the past because they are resistant to conventional chemotherapy [1] and radiation. The development of imatinib mesylate [2] a receptor tyrosine kinase inhibitor has made a major impact on the management of advanced GISTs. It specifically targets the c-*kit *(CD117) proto-oncogene gain of function mutation characterising GISTs, blocking the c-*kit *kinase, leading to growth cessation and significant durable clinical remissions. This oral drug is also active in all phases of chronic myeloid leukemia (CML) as it also targets the *bcr-abl *tyrosine kinase. It is apparent that there are sanctuary sites such as the central nervous system where imatinib does not achieve adequate concentrations. We describe the case of a man with metastatic GIST who experienced multiple cerebral relapses of disease while systemic disease progression appeared to be controlled by imatinib.

## Case presentation

A 47-year-old man presented in July 1999 with melena. A small bowel series and CT abdomen showed a jejunal mass and a 5 × 5 cm complex hepatic mass. A biopsy of the liver lesion revealed a spindle cell tumor. Laparotomy was performed, and the 7.5 cm jejunal mass was resected. A total of 4 liver lesions were noted, but not resected. The histopathology confirmed a gastrointestinal stromal tumor with clear resection margins. He subsequently had 4 cycles of attempted cytoreductive chemotherapy using doxorubicin and dacarbazine. A repeat CT scan showed progression of the liver metastases. An extended right hemihepatectomy was performed in December 1999 with successful excision of all 4 liver metastases.

The patient remained well until review in August 2001 when he complained of lethargy, right upper quadrant abdominal pain, diplopia and blurred vision in the left eye. Examination revealed weakness of the right lateral rectus ocular muscle, an amelanotic left choroidal lesion, hepatomegaly and a subcutaneous nodule in the gluteal region. An enhancing lesion in the infero-lateral region of the right globe was confirmed on CT which was thought to be the cause of the ocular muscle weakness. The CT scan also demonstrated a recurrence of multiple hepatic metastases. There were no intracerebral lesions. The gluteal lesion was biopsied and confirmed recurrence of GIST (CD117 positive). A positron emission tomogram (PET) scan also confirmed disease recurrence in the same distribution. Lumbar puncture and MRI scan of the brain excluded the presence of leptomeningeal or cerebral metastases. The patient was commenced on imatinib mesylate (STI-571, Gleevec, Novartis) 400 mg bd in September 2001 as part of a clinical trial. He felt much improved by November 2001 when a repeat CT abdomen showed stable disease but PET scan now showed no FDG uptake. By January 2002 his diplopia had completely resolved but the left-sided choroidal lesion remained unchanged on fundoscopy. The buttock lesion had also resolved.

**Figure 1:**
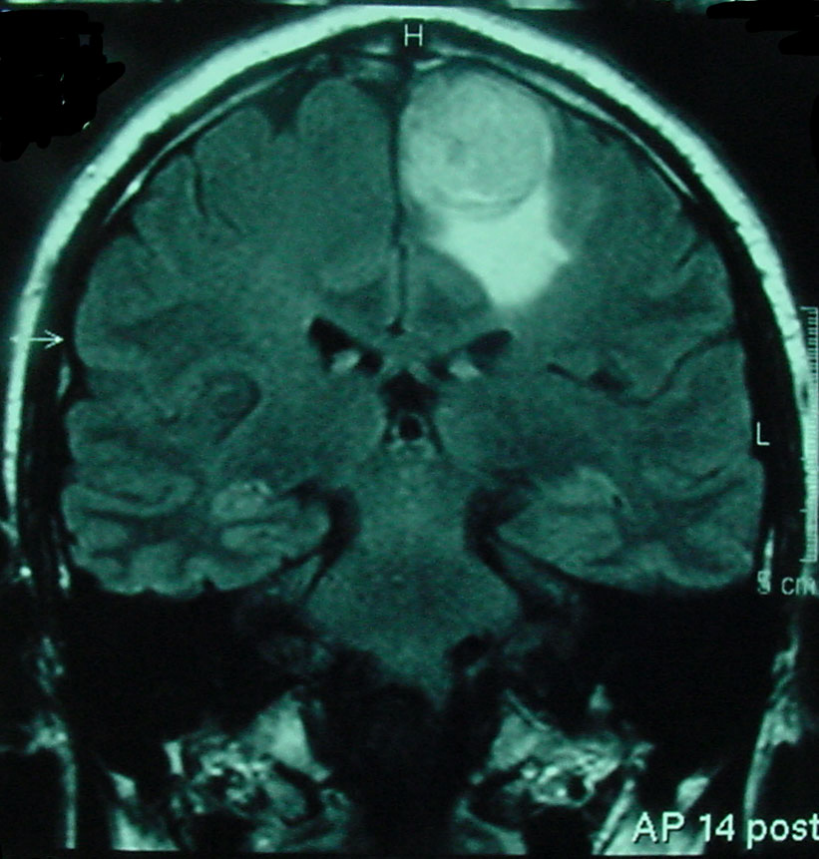
Sagittal MRI image of brain at first cerebral relapse demonstrating an enhancing left parasagittal lesion with surrounding cerebral oedema.

**Figure 2:**
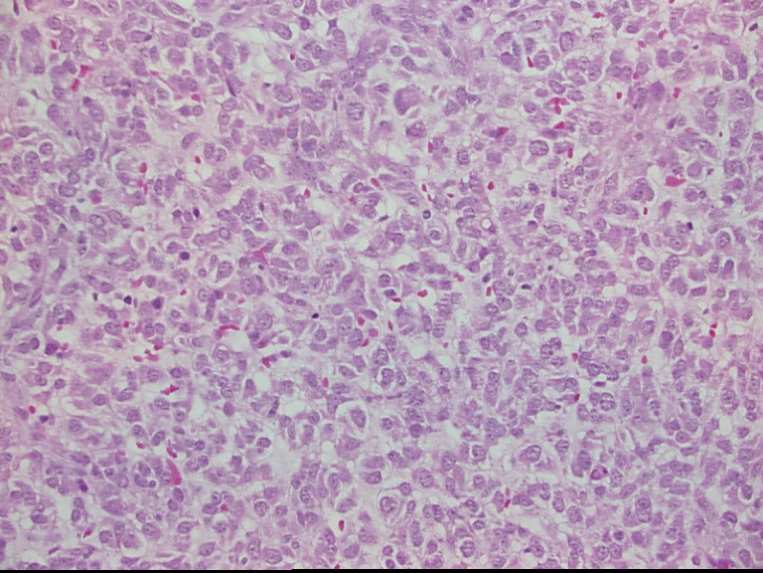
Photomicrograph of cerebral metastases showing sheets of epithelioid and spindle cells, (H&E × 200)

**Figure 3:**
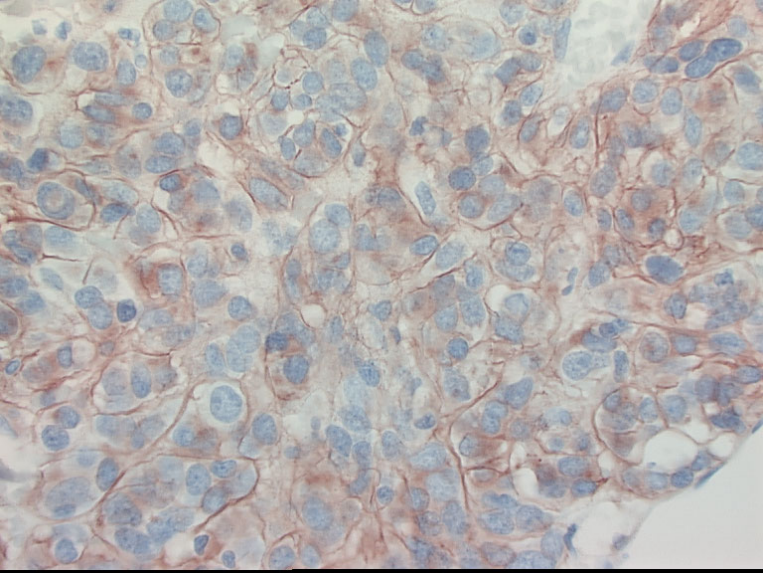
Immunohistochemical stain for CD117 (c-kit) showing staining of the cell membrane and Golgi region of the tumor cells, (× 400)

A progress CT in December 2002 showed minor enlargement of one of the liver lesions. A PET scan however continued to show no areas of abnormal FDG uptake. The patient at that time had developed a right-sided foot drop. MRI of the brain and spine demonstrated a left parasagittal tumor with radiographic features consistent with a meningioma (see Figure 1). Imatinib was ceased preoperatively as per the trial protocol. A craniotomy was performed on the 28^th ^January 2003 with complete resection of the lesion. Histopathology demonstrated metastatic GIST (CD117 positive) (see Figures 2 and 3). Post operatively the patient developed recurrent diplopia (due to recurrent right lateral rectus weakness) with blurred vision off imatinib. This was recommenced at a dose of 400 mg bd on the 14^th ^February 2003 after being ceased 6 weeks earlier because of the documented disease progression in the brain as required by the trial protocol. Drug supply was obtained through a compassionate use program. Repeat fundoscopy in February 2003 showed the choroidal lesion had enlarged. His foot drop persisted, however his diplopia again had completely resolved by March 2003.

**Figure 4:**
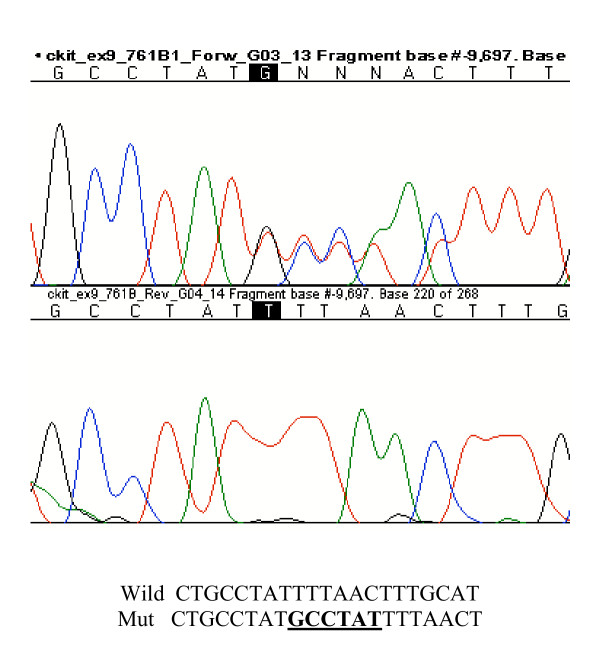
DNA sequence of part of exon 9 of c-kit for control (lower) and patient tumour (upper) samples. The patient's tumour sample contained a GCCTAT duplication

Mutational analysis on the tumor blocks was carried out. An in-frame GCCTAT insertion/duplication in exon 9 of c-*kit *in the original jejunal tumor, the liver and the cerebral metastases were detected (See Figure 4). No mutations were found in exons 11, 13 or 17 in any of the samples.

Due to limited drug supply available on compassionate use (pending local approval for reimbursement), the patient's dose of imatinib mesylate was reduced to 400 mg per day in March 2003. Six weeks later, his diplopia had returned and a progress CT abdomen demonstrated a minor progression of the liver lesions. His liver function tests remained normal. A subsequent PET scan again showed no abnormal uptake despite disease progression on the CT scan. His dose of imatinib mesylate was increased initially to 600 mg per day with resolution of the diplopia.

By May 2003 his foot drop had worsened and his dose of imatinib mesylate was increased back to 800 mg per day. Despite the increase in dose, his foot drop worsened. A repeat CT brain demonstrated a recurrence of the cerebral metastasis with surrounding vasogenic oedema in the previous site of resection. A repeat abdominal CT showed no significant change in the size of the liver metastasis and mild shrinking of the nodule in the buttock.

Due to limited treatment options available for the cerebral metastasis, a re-resection of the cranial metastasis was offered. A repeat MRI of the brain in late July 2003 confirmed the presence of the left parasagittal lesion with surrounding edema but no mass effect. Repeat craniotomy and incomplete debulking of the parasagittal metastasis was performed on the 9^th ^September 2003. A small residual area of tumor was seen on the postoperative scan. Abdominal imaging two months later showed that two of the liver lesions had increased in size with the other areas stable. He was not a candidate for further hepatic resection as there was insufficient liver reserve due to the past surgery. Radiofrequency ablation was declined as the patient did not have symptoms referable to the area.

The patient remained well until December 2003 when he experienced symptoms of headaches, worsening diplopia, right foot drop and left arm weakness. MRI scan confirmed recurrence of the cerebral metastasis with extension across the falx cerebri. He declined cranial radiotherapy treatment at the time and instead underwent a third resection of the lesion in January 2004 where incomplete debulking was achieved with early improvement of his limb weakness. In April 2004 the patient enrolled into a randomised placebo controlled clinical trial of a novel multi-kinase inhibitor SU-11248 (Pfizer) for the treatment of imatinib refractory GIST. However his condition slowly deteriorated and he died in July 2004.

## Discussion

This case illustrates a man who has had evidence of presumed ocular involvement by the GIST that initially responded to imatinib who had a cranial relapse while the systemic disease initially remained controlled. This would imply that the central nervous system could be a sanctuary site where the imatinib mesylate does not achieve adequate levels. Preclinical mice models of CML have shown development of central nervous involvement on imatinib despite systemic disease control with cerebral spinal fluid levels being 155 times lower than plasma [3]. This is supported by clinical data from treatment of CML, where isolated cerebral relapses [4,5] have been described and low cerebrospinal fluid (CSF) levels of imatinib mesylate documented during therapy with the drug [6]. One case of cerebral metastases from advanced GIST responding to imatinib mesylate has been published [7]. We would postulate that the blood brain barrier might be disrupted in some individuals thereby allowing better penetration of the drug to the brain. However in those individuals with good systemic disease control, the central nervous system may represent a sanctuary site for imatinib sensitive disease to progress.

There appears to be a relationship between the presence of different activating kinase mutations of *kit *and clinical outcomes of GISTs on imatinib [8]. The exon 9 mutation encoding the extracellular domain occurs in approximately 15% of GISTs resulting in duplication of Ala^502 ^and Tyr^503 ^This may be marker for malignant course of the disease as 71% have a highly malignant course and 59% arise exclusively in the small intestine [9]. Patients with exon 9 mutations also have a much worse prognosis than those with the more commonly found exon 11 mutations [8]. The difference is likely to be due to differences in downstream signalling in these mutations affecting the susceptibility of the GIST to imatinib and in our case may have compounded the problem with reduced CSF levels of the drug. Exon 17 mutations have been found in some GISTs that have relapsed on imatinib and are thought to be a mechanism of acquired resistance [10]. The absence of this superadded mutation would lend support to the central nervous system in being a sanctuary site in this patient.

## Conclusions

The central nervous system can be a sanctuary site to treatment of GIST with imatinib mesylate. Prolonged control of the recurrences in this location was achieved by repeated resections. This patient also highlights the importance of maintaining dosing of imatinib mesylate in the face of isolated sites of disease progression, as other metastatic sites may still be sensitive. Cessation and lowering of the dose of the drug led to temporary loss of systemic control in this case.

## Competing interests

David Goldstein and Paul Waring have both consulted and spoken on behalf of Novartis Pharma on imatinib and GIST.

## Authors' contributions

BH, DY, DG and GC participated in the clinical care of the case. PW and VB performed the mutation analysis and prepared the histopathology images. BH and DY drafted the manuscript. All authors read and approved the final manuscript.

## Pre-publication history

The pre-publication history for this paper can be accessed here:



## References

[B1] Strickland A, Letson GD, Muro-Cacho CA (2001). Gastrointestinal Stromal Tumors. Cancer Control.

[B2] Demetri GD, von Mehren M, Blanke CD, Van den Abbeele AD, Eisenberg B, Roberts PJ, Heinrich MC, Tuveson DA, Singer S, Janicek M, Fletcher JA, Silverman SG, Silberman SL, Capdeville R, Kiese B, Peng B, Dimitrijevic S, Druker BJ, Corless C, Fletcher CD, Joensuu H (2002). Efficacy and safety of imatinib mesylate in advanced gastrointestinal stromal tumors. N Engl J Med.

[B3] Wolff NC, Richardson JA, Egorin M, Ilaria R. L., Jr. (2003). The CNS is a sanctuary for leukemic cells in mice receiving imatinib mesylate for Bcr/Abl-induced leukemia. Blood.

[B4] Abruzzese E, Cantonetti M, Morino L, Orlandi G, Tendas A, Del Principe MI, Masi M, Amadori S, Orlandi A, Anemona L, Campione E (2003). CNS and cutaneous involvement in patients with chronic myeloid leukemia treated with imatinib in hematologic complete remission: two case reports. J Clin Oncol.

[B5] Petzer AL, Gunsilius E, Hayes M, Stockhammer G, Duba HC, Schneller F, Grunewald K, Poewe W, Gastl G (2002). Low concentrations of STI571 in the cerebrospinal fluid: a case report. Br J Haematol.

[B6] Takayama N, Sato N, O'Brien SG, Ikeda Y, Okamoto S (2002). Imatinib mesylate has limited activity against the central nervous system involvement of Philadelphia chromosome-positive acute lymphoblastic leukaemia due to poor penetration into cerebrospinal fluid. Br J Haematol.

[B7] Brooks BJ, Bani JC, Fletcher CD, Demeteri GD (2002). Challenges in oncology. Case 4. Response of metastatic gastrointestinal stromal tumor including CNS involvement to imatinib mesylate (STI-571). J Clin Oncol.

[B8] Heinrich MC, Corless CL, Demetri GD, Blanke CD, von Mehren M, Joensuu H, McGreevey LS, Chen CJ, Van den Abbeele AD, Druker BJ, Kiese B, Eisenberg B, Roberts PJ, Singer S, Fletcher CD, Silberman S, Dimitrijevic S, Fletcher JA (2003). Kinase mutations and imatinib response in patients with metastatic gastrointestinal stromal tumor. J Clin Oncol.

[B9] Miettenen M, Majidi M, Lsota J (2002). Pathology and diagnostic criteria of gastrointestinal stromal tumors (GISTs): a review. Eur J Cancer.

[B10] Fletcher JA, Corless CL, Dimitrijevic S, Von Mehren M, et al (2003). Mechanisms of resistance to imatinib mesylate (IM) in advanced gastrointestinal stromal tumor (GIST)[abstract]. Proc Am Soc Clin Oncol.

